# Toxicity of oxalic acid and its toxic effect on antioxidative enzymes in honey bee larvae

**DOI:** 10.17221/18/2025-VETMED

**Published:** 2025-07-03

**Authors:** Tomas Majchrak, Marek Ratvaj, Lucia Sabova, Juraj Toporcak, Ladislav Molnar

**Affiliations:** ^1^Clinic of Birds, Exotic and Free Living Animals, University Veterinary Hospital, University of Veterinary Medicine and Pharmacy in Kosice, Kosice, Slovak Republic; ^2^Department of Microbiology and Immunology, University of Veterinary Medicine and Pharmacy in Kosice, Kosice, Slovak Republic; ^3^Department of Pharmacology and Toxicology, University of Veterinary Medicine and Pharmacy in Kosice, Kosice, Slovak Republic

**Keywords:** *Apis mellifera*, detoxifying enzymes, gene expression, oxalic acid, toxicity, *Varroa destructor*

## Abstract

The production of food of plant origin is critically dependent on the pollination ability of honey bees, whose health has been deteriorating for a long time, and whose population is declining. In our *in vivo* experiment on a honey bee brood at the 4-day larval stage, we tested the following concentrations of oxalic acid: 0% (control – applied distilled water), 0.87%, 1.75%, 3.5% and 7%, corresponding to doses of 0 mg, 2.61 mg, 5.25 mg, 10.5 mg, and 21 mg of oxalic acid per dm^2^ of honeycomb with the brood. The LC_50_ values (72 h) ranged between 3.17% and 3.33%. The different LC_50_ values obtained resulted from three different methods used to calculate this indicator. The therapeutic index (TI) of oxalic acid was set to be 1.1, indicating a high risk to the honey bee brood. We observed an increased gene expression for the detoxifying enzyme glutathione-*S*-transferase (GST), but did not detect an increased gene expression for superoxide dismutase (SOD1 and SOD2), which protects the organism from oxidative stress. A decrease in gene expression was observed for prophenoloxidase and hymenoptaecin, while defensin and lysozyme did not show significant changes. These results emphasise the need for the accurate dosage and application of oxalic acid in the treatment of varroosis.

The production of food of plant origin is directly dependent on pollinators, and their ability to pollinate effectively depends on their abundance and health. However, pollinators are exposed to a wide range of negative factors, including those of anthropogenic origin, such as environmental modifications, air pollutants, plant protection products, veterinary products, as well as substances of natural origin such as mycotoxins and phytotoxins (Smith et al. 2015).

Among the dominant pollinators of plants are honey bees (*Apis mellifera*). Beekeeping worldwide shows a long-term declining trend, which can be attributed to the high honey bee morbidity due to parasitic, bacterial, and viral diseases and environmental pollution. Quantifying the relative impact of different chemicals on honey bee health compared to other stressors, such as varroosis, bacterial infections, viruses, and nutrition, was identified as a priority to support the development of comprehensive risk assessment methods (Carnesecchi et al. 2019).

*Varroa destructor* is currently the most important parasite of the honey bee and the main cause of colony mortality. The increasing resistance of this parasite to traditional treatments highlights the need for intensive research on new methods to combat varroosis (Bahreini et al. 2020).

Medications used to treat varroosis are divided into four main groups. The first group consists of synthetic pyrethroids, such as flumethrin and tau-fluvalinate. The second group consists of essential oils, the third of organic acids, and the fourth of amitraz, a non-systemic acaricide and insecticide. Each of these groups has specific properties and mechanisms of action (Toporcak et al. 2020).

An essential requirement for the registration and use of drugs is the determination of their toxicity to the target organism (parasite) as well as their toxicity to the treated individual, in this case, the honey bee and its brood. Prospective drugs for the treatment of varroosis include low-molecular-weight organic acids such as formic acid, oxalic acid, and lactic acid (Girisgin and Aydin 2010).

In toxicity tests, monitoring changes in the superoxide dismutase (SOD) activity is essential when assessing the cellular damage in the test organism. SOD is an extremely valuable antioxidant that helps protect cells from destruction. The substrate of SOD is the superoxide radical anion (O_2–_), which is formed by the transfer of one electron to molecular oxygen. This radical is responsible for direct damage to biological macromolecules and the formation of other reactive oxygen species. SOD maintains the concentration of superoxide radicals at a low level, thus playing a key role in defence against oxidative stress (Fridovich 1997).

Glutathione-transferases are known to protect against oxidative stress. Primary antioxidant enzymes play a largely preventive role, degrading reactive oxygen species (ROS) and thereby preventing damage to cellular components, while also initiating lipid peroxidation. In the case of ROS-induced lipid peroxidation, secondary defence enzymes are involved in the removal of lipid hydroperoxides (LOOH), thereby terminating the autocatalytic chain of lipid peroxidation and protecting cell membranes.

Glutathione peroxidase (GPx) and glutathione-*S*-transferase (GST), which catalyse the glutathione-dependent reduction of LOOH through their peroxidase activity, are the major secondary defence against ROS-induced lipid peroxidation. Glutathione reductase plays an important role in cellular antioxidant protection. This enzyme catalyses the regeneration of GSH, a substrate for GPx (Alghazal et al. 2008).

The enzymes present in honey bees in the form of superoxide dismutases (SOD1, SOD2), catalase (CAT), and glutathione-*S*-transferase (GST) protect the honey bee organism from free radicals (Corona and Robinson 2006). The production of free radicals is a physiological process, but their overproduction can occur due to external factors, such as pesticides (He et al. 2021), electromagnetic radiation (Migdal et al. 2021), or seasonal changes (Orcic et al. 2017).

Dismutases act in the first step of defence against free radicals and convert the superoxide radical into oxygen and hydrogen peroxide. SOD1 acts in the cytoplasm, while SOD2 acts in the mitochondria. CAT acts in the second step, breaking down hydrogen peroxide into water and oxygen. Hydrogen peroxide is also broken down by GST, which, however, has a detoxifying effect as well (Fukui and Zhu 2010).

The immune system of honey bees consists of humoral and cellular components, similar to those of mammals, but is much simpler. The humoral immune response is primarily mediated by antimicrobial peptides (Danihlik et al. 2015). Concentrations of lysozyme and hymenoptaecin, which belong to this group, are physiologically elevated in infected larvae (Chan et al. 2009).

Our experimental study aimed to determine the toxicity of oxalic acid to young honey bee larvae under *in vivo* conditions and to analyse changes in the relative expression of the genes responsible for detoxification and the immune response in honey bee larvae, including the immune response.

## MATERIAL AND METHODS

### Determination of the toxicity of oxalic acid in honey bee larvae

To test the toxicity of oxalic acid in honeybee larvae, we employed a modified methodology by Sabova et al. (2019). This involved the selection and rearing of suitable honey bee colonies and the subsequent selection of suitable larvae under *in vivo* conditions.

**Figure 1:**
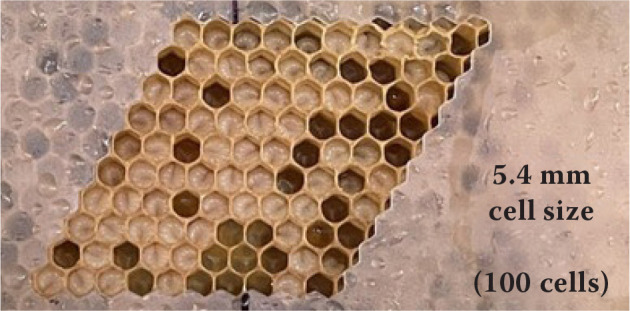
Original application template (1 dm^2^, one hundred honey bee cells/larvae)

Currently registered veterinary drugs against varroosis based on oxalic acid recommend a therapeutic dose of 0.3 ml of a 3% solution per dm^2^ of honeycomb (EMA 2017; EMA 2018). In our *in vivo* experiment on the honey bee brood at the 4-day larval stage, we tested the following concentrations of oxalic acid (VWR BDH Prolabo^®^ Chemicals, Leuven, Belgium): 0% (control – applied distilled water), 0.87%, 1.75%, 3.5% and 7%, corresponding to doses of 0 mg, 2.61 mg, 5.25 mg, 10.5 mg, and 21 mg of oxalic acid per dm^2^ of honeycomb with the brood. One dm^2^ of honeycomb contains an average of 100 cells, as shown in Figure 1. The number of larvae in the tested area of 1 dm^2^ differed in each case. The individual doses of oxalic acid per honeycomb cell are as follows: control, 26.1 μg, 52.5 μg, 105 μg, and 210 μg per cell in a spray form. The recommended dose of 0.3 ml per dm^2^ of brood comb was applied in this experiment.

To ensure that the larvae in the experiment were four days old at the time of application, the queens needed to be confined in their colony in an exclusion cage containing an empty comb – Organisation for Economic Co-operation and Development 237 (OECD 2013). We followed the guidelines outlined in OECD 237 (OECD 2013). After synchronisation of the larvae, a plastic sheet for the hygiene test containing 100 cells of 5.4 mm was used. Three applications of the solution were made to three locations within one hive. The solution, preheated to 37.5 °C, was applied at a right angle (90°) from a distance of 25 cm from the honeycomb. The control larvae were sprayed with distilled water. The larval mortality was checked and recorded 72 h (3 days) after application. The mortality data are expressed as the number of dead larvae and as adjusted percentages, following the guidelines (OECD 2013; Sabova et al. 2019).

The following mathematical programs and methods were used to calculate toxicity: ToxRat Professional v3.3.0, Finney’s Probit Analysis, and the interpolation method of Roth et al. (1962).

### Determination of the relative gene expression of the detoxifying enzymes

To determine the relative gene expression, the larvae were homogenised in two steps due to their high volume. First, the honey bee larvae were mechanically homogenised in a tube using a pestle. A portion of this homogeneous mass was then transferred to a tube containing 1 ml of TRIzol (Thermo Fisher Scientific, Waltham, USA) and further homogenised using 1.4 mm diameter zirconia beads (Bertin Technologies, Montigny-le-Bretonneux, France) on a Precellys 24 homogeniser (Bertin Technologies, Montigny-le-Bretonneux, France). The homogenisation was performed at 4 500 rpm with the cycle consisting of two 30-second phases separated by a 15-second break.

RNA was isolated from the prepared homogenate according to the TRIzol manufacturer’s protocol. The RNA quality and quantity were assessed spectrophotometrically using a NanoDrop 8000 instrument (Thermo Fisher Scientific, Waltham, USA). From each sample, 1 000 ng of RNA was collected as a template for the cDNA synthesis using the commercial QuantiTect Reverse Transcription Kit (QIAGEN, Hilden, Germany). This protocol also included the removal of gDNA from the samples. The resulting cDNA was stored at –20 °C until further analysis.

The relative gene expression was determined in the samples by quantitative polymerase chain reaction (qPCR) on a Bio-Rad CFX96 instrument (Bio-Rad Laboratories, Hercules, USA). The reaction mixture had a total volume of 10 μl and contained: 5 μl of the Luna Universal qPCR Master Mix (New England Biolabs, Ipswich, MA, USA), 0.3 μl of a forward and reverse primer (c = 10 μM/μl), 4 μl cDNA (c = 10 ng/μl), and deionised water to make up the volume. The samples were analysed in duplicate. β*-actin* and *rp49* were used as the reference genes; the primer sequences are listed in Table 1.

**Table 1:** List of the used primers

Gene	Sequence (5'–3')	Reference
*Catalase* (*cat*)	F: GGCGGCTGAATTAAGTGCTA	Collins et al. (2004)
	R: TTGCGTTGTGTTGGAGTCAT	
*Superoxide dismutase* (*sod1*)	F: AGCAGATGCAAGTGGTGTTG	Collins et al. (2004)
	R: GAGCACCAGCATTTCCTGTAG	
*Superoxide dismutase* (*sod2*)	F: GTCGCCAAAGGTGATGTCAATAC	Li et al. (2014)
	R: CGTCTGGTTTACCGCCATTTG	
*Glutathione-S-transferase 1* (*gst-1*)	F: AGGAGAGGTGTGGAGAGATAGTG	Li et al. (2014)
	R: CGCAAATGGTCGTGTGGATG	
*Defensin 1* (*def-1*)	F: TGTCGGCCTTCTCTTCATGG	Li et al. (2016)
	R: TGACCTCCAGCTTTACCCAAA	
*Lysozyme* (*lys*)	F: CCAAATTAACAGCGCCAAGT	Evans et al. (2006)
	R: GCAATTCTTCACCCAACCAT	
*Hymenoptaecin* (*hym*)	F: CTCTTCTGTGCCGTTGCATA	Evans et al. (2006)
	R: GCGTCTCCTGTCATTCCATT	
*Prophenoloxidase activator* (*ppoa*)	F: GTTTGGTCGACGGAAGAAAA	Evans et al. (2006)
	R: CCGTCGACTCGAAATCGTAT	
*Beta-actin* (β*-act*)	F: TGCCAACACTGTCCTTTCTG	Scharlaken et al. (2007)
	R: AGAATTGACCCACCAATCCA	
*Ribosomal protein 49* (*rp49*)	F: AAGTTCATTCGTCACCAGAG	de Miranda and Fries (2008)
	R: CTTCGAGTTCCTTGACATTATG	

The PCR protocol consisted of the following steps: initial denaturation at 95 °C for 60 s, followed by 40 cycles of denaturation at 95 °C for 15 s and hybridisation with extension at 60 °C for 30 seconds. A melting curve analysis was performed to confirm the amplification of the specific product.

The gene expression results were evaluated by the 2^−ΔΔ*CT*^ method relative to the reference genes. A statistical analysis was performed using the JASP software (v0.19.2) and one-way analysis of variance (ANOVA), supplemented with Tukey’s post-hoc test.

## RESULTS

The toxic effects of the observed concentrations of oxalic acid on the honey bee larvae were evaluated under *in vivo* conditions after spraying. The assessment was conducted in accordance with OECD Guideline 237 (OECD 2013). The average mortality rate of the honey bee larvae brood at the different application concentrations of oxalic acid is presented in Table 2.

**Table 2:** Mortality (72 h) of the honey bee larvae after the application of different concentrations of oxalic acid (three applications)

Test concentration (% of oxalic acids)	No. of cells tested	Average number of live larvae before application	Average number of dead larvae after application	% Mortality (72 h)	±SD
0 (control group)	100	68.33	1.67	2.41	0.45
0.87	100	50.33	5.67	10.94	2.68
1.75	100	68.00	24.50	36.21	15.92
3.50	100	80.50	41.50	51.54	19.30
7.00	100	56.33	44.00	79.39	17.25

**Figure 2:**
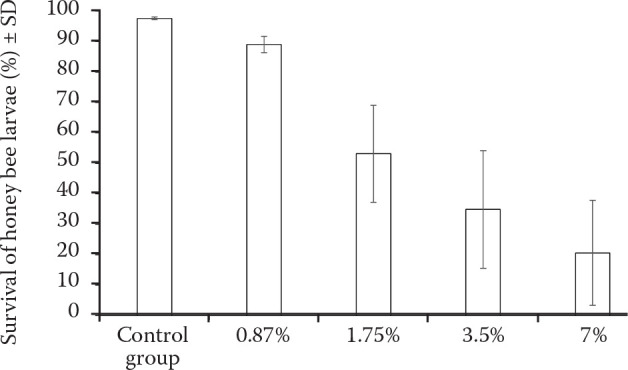
Survival of the honey bee larvae 72 h after the application of different concentrations of the oxalic acid solution

As shown by the results, the highest mortality percentage of honey bee larvae after 72 h was observed with the application of a 7% oxalic acid concentration, where an average mortality of 79.39% was recorded among the honey bee larvae. On the contrary, the lowest mortality percentage after 72 h was recorded at the 0.87% oxalic acid concentration, with an average mortality of 10.94%. In the control group, the average mortality was 2.41%. The average percentage survival of the honey bee larvae in our experiment in the control group after 72 h and the experimental groups is shown in Figure 2.

**Figure 3:**
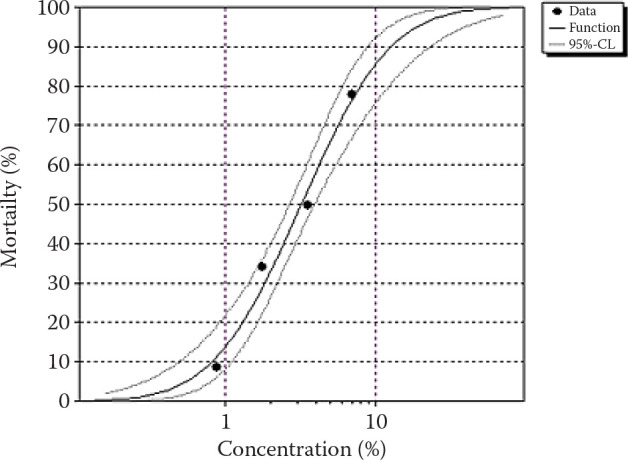
Mortality curve of the honey bee larvae as related to the oxalic acid concentration (ToxRat Professional v3.3.0)

The median lethal concentration (LC_50_) of the oxalic acid solution was calculated using ToxRat Professional v3.3.0. Finney’s Probit Analysis and the method according to Roth et al. (1962) were simultaneously used to calculate the LC_50_. Figure 3 presents the curves and 95% confidence interval calculated by ToxRat Professional v3.3.0. The statistical significance value for the LC_50_ (72 h) calculation is *P*(*F*) ≤ 0.05. The LC_50_ range values after a single spray of oxalic acid solution for *A. mellifera* larvae are between 3.17% (ToxRat Professional v3.3.0) and 3.33% (Finney’s Probit Analysis). The LC_50_ (72 h) for young larvae after a single spray of oxalic acid solution, calculated according to Roth et al. (1962), is 3.26% (Table 3).

**Table 3:** Toxicity of the oxalic acid solution to the honey bee larvae

Method	LC_50_ (72 h) (percent of solution)	Confidence interval (percent of solution)
ToxRat Professional v3.3.0	3.17	2.65–3.89
Finney’s Probit Analysis	3.33	2.12–5.23
Roth et al. (1962)	3.26	2.07–5.13

**Figure 4:**
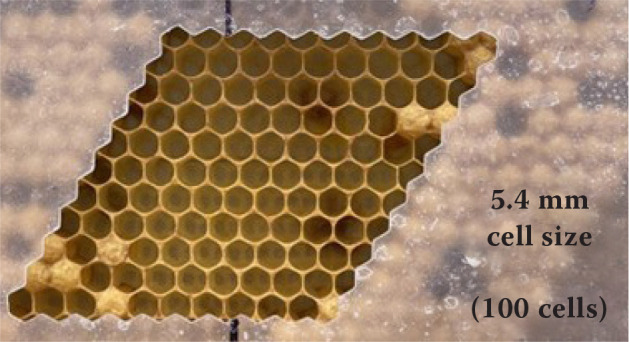
Toxic effect of the 7% oxalic acid on the honey bee brood 72 h after the single exposure under the *in vivo* experimental conditions

The LD_50_ of oxalic acid for a single honey bee larva was calculated based on the obtained LC_50_ (72 h) values of this acid, the applied dose per 1 dm^2^, and the known number of honeycomb cells to which the acid was applied. The LD_50_ values (μg/larva) of oxalic acid, with the 95% confidence intervals obtained by the methods used, are given in Table 4. Figure 4, for demonstration, shows the toxic effect of the 7% oxalic acid application on the honey bee brood after 72 h of exposure *in vivo*.

**Table 4:** Toxicity (72 h) of the oxalic acid to the young honey bee larva (4-day-old)

Method	LD_50_ (μg/larva)	Confidence interval (μg/larva)
Roth et al. (1962)	97.8	62.1–153.9
Finney’s Probit Analysis	99.9	63.6–156.9
ToxRat Professional v3.3.0	95.1	79.5–116.7

**Figure 5:**
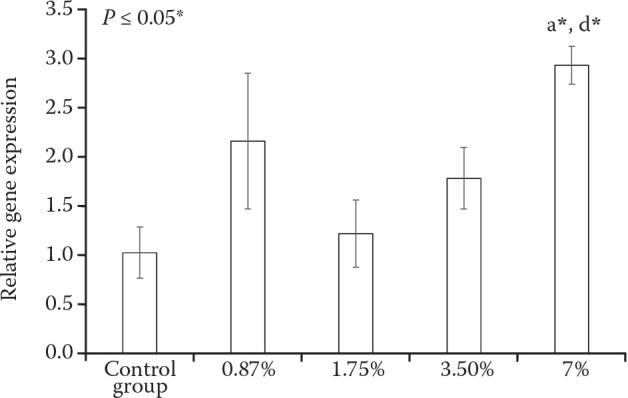
Relative gene expression of *gst* Significant difference compared to: a = control, and d = 1.75%

After applying different concentrations of oxalic acid to the honey bee brood, we did not observe statistically significant increases in the expression of the *CAT*, *SOD1,* and *SOD2* genes in the experimental larval groups compared to the control, nor between the groups relative to each other. In summary, the expression results of the detoxification genes after application of different acid concentrations are shown in Table 5. For the *GST* gene, we observed increased expression. There was a significant increase in the expression of the *GST* gene when 7% oxalic acid was applied compared to the control group and to the group of larvae to which 1.75% oxalic acid was applied (Figure 5).

**Table 5:** Relative gene expression of the selected genes

Gene	Control (0%)	0.87%	1.75%	3.5%	7%
*cat*	1.012 ± 0.158	1.343 ± 0.108	1.11 ± 0.087	1.443 ± 0.171	1.204 ± 0.275
*sod1*	1.012 ± 0.15	1.357 ± 0.414	0.905 ± 0.233	1.047 ± 0.173	1.237 ± 0.124
*sod2*	1.04 ± 0.282	1.031 ± 0.123	0.801 ± 0.13	1.045 ± 0.134	0.909 ± 0.063
*gst*	1.03 ± 0.261	2.162 ± 0.690	1.225 ± 0.341	1.787 ± 0.313	2.934 ± 0.193^a^*^,d^*
*def*	1.509 ± 0.942	5.626 ± 1.679^a^*	0.620 ± 0.259^b^*^,c^**	2.576 ± 0.737^c^**	7.813 ± 0.374^a^**
*lys*	1.095 ± 0.429	1.885 ± 0.644	1.227 ± 0.046	2.329 ± 0.709	2.502 ± 0.658
*hym*	1.250 ± 0.642	1.576 ± 0.275	0.647 ± 0.002	1.918 ± 0.587	1.819 ± 0.184
*ppoa*	1.094 ± 0.423	0.833 ± 0.085	0.884 ± 0.099	1.654 ± 0.302^b^*	1.476 ± 0.124

*P ≤ 0.05; **P ≤ 0.01

Results calculated as 2^−ΔΔ*CT*^ and presented with the standard deviation; Significant difference compared to a = control, b = 0.87%, c = 7%, d = 1.75%

**Figure 6:**
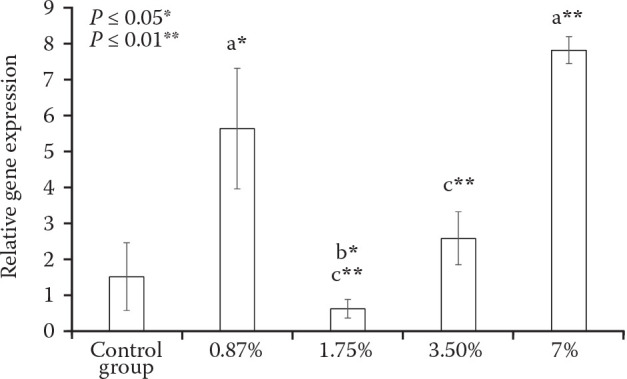
Relative gene expression of *def-1* Significant difference compared to: a = control, b = 0.87% and c = 7%

**Figure 7:**
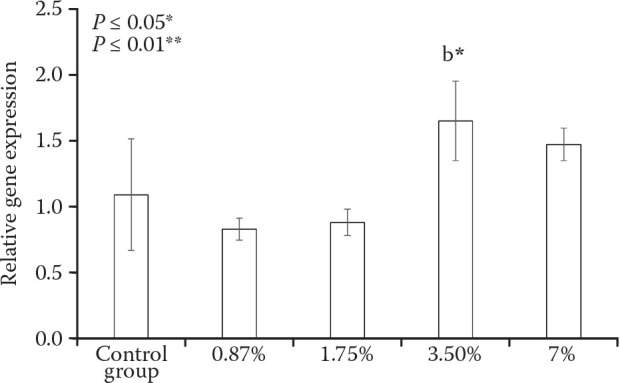
Relative gene expression of *ppoa* Significant difference compared to b = 0.87%

To evaluate the stimulation of the larval immune system following the single oxalic acid spray application, we monitored the relative gene expression of *DEF, LYS HYM,* and *PPOA*. No statistically significant change was observed for the antimicrobial peptides encoded by the *LYS* and *HYM* genes. However, significant changes in the expression of *DEF* and *PPOA* were observed. The relative gene expression of *DEF* was significantly increased at the oxalic acid concentrations of 0.87% and 7% compared to the control group. The group receiving 1.75% oxalic acid showed significantly lower expression compared to the 0.87% and 7% concentration groups. The group exposed to 3.5% oxalic acid had a significantly reduced relative expression of *DEF* compared to the 7% concentration group (Figures 6 and 7).

The relative gene expression of *PPOA* was significantly increased in the 3.5% acid group compared to the 0.87% acid group (Figure 7).

## DISCUSSION

The production of food of plant origin is critically dependent on pollinators, including honey bees, whose pollination ability depends on their population size and health. Globally, the number of honey bee colonies has been declining for a long time, primarily due to environmental pollution and high honey bee morbidity. One of the most serious parasites of honey bees (*A. mellifera*) is currently the *V. destructor* mite, whose resistance to acaricides poses a major challenge. Consequently, developing new integrated pest management control procedures for *Varroa destructor* remains a major focus of scientific interest (Rosenkranz et al. 2010; Goulson et al. 2015).

The acaricide amitraz has long been used to treat varroosis. In recent years, this has created strong selection pressure on *V. destructor* mite populations. This pressure has contributed to the development of resistance to amitraz. Mutations N87S and Y215H in the β2-adrenergic-like octopamine receptor (Octβ2R), the target site of amitraz, have been identified as causes of this resistance (Hernandez-Rodriguez et al. 2025).

Therefore, low-molecular-weight organic acids (formic acid, lactic acid, and oxalic acid), or their combinations with amitraz or coumaphos, have been introduced to treat varroosis. These therapies have involved monitoring the effect of oxalic acid on *Varroa destructor* mites while evaluating its effect on honey bees under *in vitro* and *in vivo* conditions. A 4% oxalic acid in sugar solution demonstrated up to 93.7% efficacy on the *Varroa destructor* when applied in autumn. No reductions in the queen bee’s egg-laying or adult honey bee deaths due to the treatment were observed during the experiments (Girisgin and Aydin 2010).

The LC_50_ (72 h) value of the oxalic acid solution, determined by us and applied in spray form to a honey bee (*A. mellifera*) brood under *in vivo* conditions, calculated using various methods – ToxRat Professional v3.3.0., Finney’s Probit Analysis, and the method according to Roth et al. (1962) – ranged from 3.17% to 3.33% (Table 3). Using the OECD 237 methodology Sabova et al. (2019) determined the LC_50_ (72 h) value of an oxalic acid solution applied in spray form to a honey bee brood under *in vitro* conditions. The LC_50_ (72 h) value found was 2.425%. Our slightly higher LC_50_ (72 h) value can be explained by the fact that our experiments were carried out under *in vivo* conditions, where the hygienic behaviour capacity of honey bees plays a significant role in the application of chemicals. Honey bees can metabolise or excrete toxic substances, reducing their effectiveness and leading to a higher tolerance of the organism. This explains the higher LC_50_ values compared to the *in vitro* conditions.

It is also similar in the case of the determination of the LD_50_ for spray application to the honey bee larvae. In our experiment, we determined LD_50_ (72 h) ranging from 95.1 to 99.9 μg per larva under *in vivo* conditions (Table 4). The authors Sabova et al. (2019) reported an estimated LD_50_ (72 h) of 45 μg for a honey bee larva. The differences between our results and those of Sabova et al. (2019) highlight the importance of experimental conditions (*in vivo* vs *in vitro*) when assessing the toxicity of chemicals to honey bees. The higher LD_50_ values under *in vivo* conditions can again be explained by the metabolic activity and hygienic behaviour of honey bees, which contribute to the reduced substance toxicity. These differences between the *in vivo* and *in vitro* conditions highlight the importance of considering the biological mechanisms of bees when assessing the toxicity of chemicals.

In a feeding toxicity study, the LC_50_ of oxalic acid was determined for a honey bee brood. Oxalic acid was administered at different concentrations in a diet containing 50% royal jelly and 50% sucrose. The LC_50_ values ranged from 0.649% to 0.959%, depending on the brood age (Terpin et al. 2019). The high toxicity observed after oral administration can be explained by damage to the larval gut epithelium. Gregorc et al. (2004) have shown that necrotic damage to 25% of the epithelial cells occurs as early as 5 h after the oral application of a 2.97% oxalic acid solution in a 31.97% sucrose solution. It follows from the above that although treating honey bees against varroosis with the recommended dose of 5 ml of 3–5% oxalic acid in a sugar solution (applied by trickling) is safe for adult bees, it poses a high risk to the honey bee brood. Therefore, applying oxalic acid by trickling in the bee colonies is particularly suitable during the winter broodless period. For this reason, a spray application of oxalic acid is significantly safer as it minimises the risk to the honey bee brood.

The therapeutic index (TI) provides information on the relative safety of a drug. This value is given as the ratio between the median toxic dose and the median therapeutic dose of the drug. Safe drugs have TI values in the tens to hundreds. Our calculations show a TI value of 1.1 for oxalic acid for the honey bee larvae. A low TI value of oxalic acid indicates a high risk to the honey bee brood. Therefore, the precise preparation of the oxalic acid application solution and the correct dosage on the brood comb are essential in practice.

The effect of oxalic acid on *Varroa destructor* is due to the mite’s high sensitivity to the oxalic acid – the LD_50_ (24 h) of oxalic acid after spray application is 5.12 μg per *Varroa destructor* under *in vitro* conditions (Aliano et al. 2006). The LD_50_ (72 h) value determined by us for the spray application to the honey bee larvae ranges from 95.1 μg to 99.9 μg per larva under *in vivo* conditions. Thus, the toxicity of oxalic acid to *Varroa destructor* is 20 times higher than to the honey bee larvae. Due to the toxicity of oxalic acid to the brood, the application method and dosage must be carefully considered to minimise any negative effects on the colony.

The toxic effect of oxalic acid on the honey bee brood demonstrates its biological effect. As shown by the results of Qi et al. (2020), honey bee larvae are sensitive to chemical exposure, and high doses can increase the production of antioxidant enzymes. Oxalic acid, commonly used to control varroosis, is generally considered safe for bees. In our experiment, we did not observe the increased expression of *SOD1, SOD2* or *CAT* genes in the exposed larvae. This suggests that, at a given concentration, oxalic acid does not stimulate the honey bees’ antioxidant system. This lack of activation of the antioxidant response could weaken the organisms of the larvae or increase their mortality (Sagona et al. 2024).

In our study, the *GST* gene expression was significantly affected only at high concentrations of oxalic acid, 7% (Figure 5). This enzyme is usually associated with exposure to xenobiotics, particularly pesticides (Li et al. 2017; Decio et al. 2021), and its activity increases in such cases. Thus, our results suggest that the use of oxalic acid in colonies is safe as long as the concentration does not exceed the level that activates the natural detoxification response in larvae.

The immune system of honey bees, although simpler than that of mammals, consists of humoral and cellular immunity. The humoral immune response is mainly provided by antimicrobial peptides (AMPs) (Danihlik et al. 2015), including lysozyme and hymenoptaecin, the levels of which are physiologically elevated in the infected larvae (Chan et al. 2009). In our study, we observed no change in the relative expression of these antimicrobial peptides, which is consistent with the study of Boncristiani et al. (2012). This may be due to a different mechanism of stimulation of AMP synthesis during bacterial infection.

On the other hand, synthetic acaricides, such as flumethrin, fluvalinate, and amitraz, used in beekeeping, increased the hymenoptaecin expression, but did not affect the lysozyme or defensin expression (Garrido et al. 2013). Regarding defensin, in our study, we observed an increased relative expression in the group exposed to the highest oxalic acid concentration (7%), but, paradoxically, also in the group exposed to the lowest oxalic acid concentration (0.87%) (Figure 6). This may be due to the individual variability in the defense in expression, which varies considerably between individuals. Thus, in larvae, the immune response was stimulated.

Within the group of larvae treated with 1.75% oxalic acid, we observed reduced *DEF* expression*.* It would be detrimental to both larvae and adult bees if the product used to treat them reduced their immune response. The aforementioned synthetic acaricides, as well as oxalic acid, are used to treat honey bees infested with *Varroa destructor* that transmits a number of pathogens (Kang et al. 2016). Potentially, an immunosuppressed individual would have a more difficult time defending itself against these pathogens, leading to its death. Moreover, the immune system of honey bees develops with age; a stronger immune response is observed in adult honey bees (Wilson-Rich et al. 2008), which increases the infection risk in developing larvae.

The phenoloxidase cascade, which leads to melanisation, is one of the basic defence mechanisms of invertebrates against microorganisms. This biochemical process involves several successive reactions. Upon recognition of a pathogen, prophenoloxidase is activated by the prophenoloxidase-activating enzyme (PPOA) (Ashida and Brey 1998). In our study, we observed no change in the *PPOA* gene expression compared to the control group (Figure 7). This result can be viewed as positive because melanisation is critical not only for the immune response, but also for bee exoskeleton formation (Sugumaran 2022). The disruption of this process could lead to developmental or colour abnormalities (Bitondi et al. 1998; Liu et al. 2015), as well as a weakened immune response (Luz et al. 2022).

Similar selective changes in antioxidant enzyme activity in homogenates of whole surviving larvae were reported by Sabova et al. (2019) at the same doses of oxalic acid. The specific activities of superoxide dismutase and catalase showed similar trends, depending on the oxalic acid concentration. At the lowest oxalic acid concentration (0.87%), the authors have not observed any changes in the activity of these enzymes. A slight activation of the enzymes (approximately 1.5-fold) was observed at the 1.75% oxalic acid concentration. With a further increase in the oxalic acid concentration, the enzyme activity gradually decreased. The specific glutathione-*S*-transferase (GST) activity increased significantly at the 3.5% oxalic acid concentration (1.0 U/mg ± 0.2 U/mg) compared to the control group (0.66 U/mg ± 0.03 U/mg) and the 0.87% oxalic acid group (0.547 U/mg ± 0.003 U/mg). At this oxalic acid concentration (3.5%), a statistically significant increase in the level of the biochemical marker of oxidative stress, TBARS (Thiobarbituric Acid Reactive Substances), was observed, which was more than 1.7 times higher compared to the control group.

The obtained results show that oxalic acid is safe for adult bees at a therapeutic dose of 0.3 ml of a 3% solution per dm^2^ of honeycomb, but poses some toxicological risk to young honey bee larvae. This risk is supported by the low therapeutic index value (TI = 1.1), indicating a narrow safety margin between efficacy and toxicity. In addition, oxalic acid may inhibit the larvae’s immune response, increasing their susceptibility to infections. Therefore, it is essential in the treatment of varroosis to strictly follow the recommended concentration (3%) and dosage (0.3 ml per dm^2^) of oxalic acid in spray applications to minimise the risk of brood intoxication. These precautions are key to maintaining the health of colonies and maintaining their pollination ability, which is essential for agricultural production.
